# DNA damage signaling regulates age-dependent proliferative capacity of quiescent inner ear supporting cells

**DOI:** 10.18632/aging.100668

**Published:** 2014-05-21

**Authors:** Maarja Laos, Tommi Anttonen, Anna Kirjavainen, Taija af Hällström, Marikki Laiho, Ulla Pirvola

**Affiliations:** ^1^ Department of Biosciences, 00014 University of Helsinki, Finland; ^2^ Institute for Molecular Medicine Finland FIMM, 00014 University of Helsinki, Finland; ^3^ Department of Radiation Oncology and Molecular Radiation Sciences, Johns Hopkins University School of Medicine, Baltimore, MD 21287, USA

**Keywords:** DNA damage, DNA repair, cell cycle re-entry, regeneration, supporting cell, hair cell, inner ear

## Abstract

Supporting cells (SCs) of the cochlear (auditory) and vestibular (balance) organs hold promise as a platform for therapeutic regeneration of the sensory hair cells. Prior data have shown proliferative restrictions of adult SCs forced to re-enter the cell cycle. By comparing juvenile and adult SCs in explant cultures, we have here studied how proliferative restrictions are linked with DNA damage signaling. Cyclin D1 overexpression, used to stimulate cell cycle re-entry, triggered higher proliferative activity of juvenile SCs. Phosphorylated form of histone H2AX (γH2AX) and p53 binding protein 1 (53BP1) were induced in a foci-like pattern in SCs of both ages as an indication of DNA double-strand break formation and activated DNA damage response. Compared to juvenile SCs, γH2AX and the repair protein Rad51 were resolved with slower kinetics in adult SCs, accompanied by increased apoptosis. Consistent with the *in vitro* data, in a *Rb* mutant mouse model *in vivo*, cell cycle re-entry of SCs was associated with γH2AX foci induction. In contrast to cell cycle reactivation, pharmacological stimulation of SC-to-hair-cell transdifferentiation *in vitro* did not trigger γH2AX. Thus, DNA damage and its prolonged resolution are critical barriers in the efforts to stimulate proliferation of the adult inner ear SCs.

## INTRODUCTION

Sensory hair cells and supporting cells (SCs) of the cochlear and vestibular organs are highly differentiated cells that do not proliferate at adulthood. Because mammalian hair cells do not regenerate, their loss following trauma leads to permanent functional (hearing and balance) deficits. SCs hold promise as a platform for therapeutic hair cell regeneration, analogous to natural regeneration in the non-mammalian inner ears in which SCs divide and transdifferentiate into hair cells [1]. In the field of regenerative medicine, stimulation of cellular reprogramming and expansion of quiescent cells have been shown to cause genetic abnormalities and cell death, for example in response to DNA damage triggered by ectopic oncogene expression [2, 3]. We have previously shown that, upon forced cell cycle re-entry, SCs of the adult utricle, one of the vestibular organs, accumulate DNA damage and arrest in the cell cycle. We hypothesized that the limited capacity of adult SCs to cope with DNA damage underlies the restrictions in completing normal cell cycles [4].

Oncogene overexpression and genotoxic agents cause several types of DNA lesions, including DNA double-strand breaks (DSBs) that represent the most lethal form of DNA damage. The activated DNA damage response (DDR) orchestrates damage detection and repair. DDR activates cell cycle checkpoints to guard against inappropriate cell cycle progression until DNA lesions are resolved. If DNA damage remains unresolved, the outcome is cell cycle arrest or death [5]. Activation of the proximal components of the DDR pathway, the members of a family of phosphoinositide-3-kinase-related protein kinases (PIKKs), feeds to their downstream effectors, including the histone H2A variant H2AX. It is activated by PIKK phosphorylation on serine 139, termed as γH2AX. γH2AX accumulates in megabase regions surrounding DSBs, resulting in the formation of foci that act as docking sites for other DDR components [6, 7]. γH2AX foci can be detected by antibodies and used as indicators for the presence of DNA damage [6]. The rapid γH2AX activation after damage and the resolution of the foci after repair have suggested that γH2AX can be used as a marker for monitoring cellular DDR [8, 9].

Compared to proliferating cells, DNA damage signaling in postmitotic cells is poorly studied, especially in connection with cell cycle manipulation. Upon forced proliferation, γH2AX induction has been shown in neurons of the central nervous system and in the inner ear hair cells, and was found to lead to apoptosis of these highly differentiated cells [10, 11]. Upon DNA damage triggered by genotoxic agents, activation of other components of the DDR pathway, besides H2AX, has been shown in postmitotic neurons and epithelial cells [12-14], but, surprisingly, not in astrocytes, even though these cells were found to be DNA repair-proficient [9]. We have here focused on DNA damage signaling in the mouse inner ear SCs. These glial-like cells show greater proliferative plasticity compared to hair cells of the same tissue. SCs can proliferate neonatally, but thereafter this plasticity is rapidly lost [15, 16]. We have comparatively studied DNA damage signaling in the juvenile and adult SCs triggered to re-enter the cell cycle through ectopic cyclin D1 (cD1) expression. We demonstrate age-dependent differences in DNA damage resolution and repair, suggesting that DDR in the adult SCs represents a critical element in the attempts to stimulate proliferative regeneration as an approach for treatment of hearing and balance impairments.

## RESULTS

### Adenoviral tropism to postnatal inner ear supporting cells

**Figure 1:**
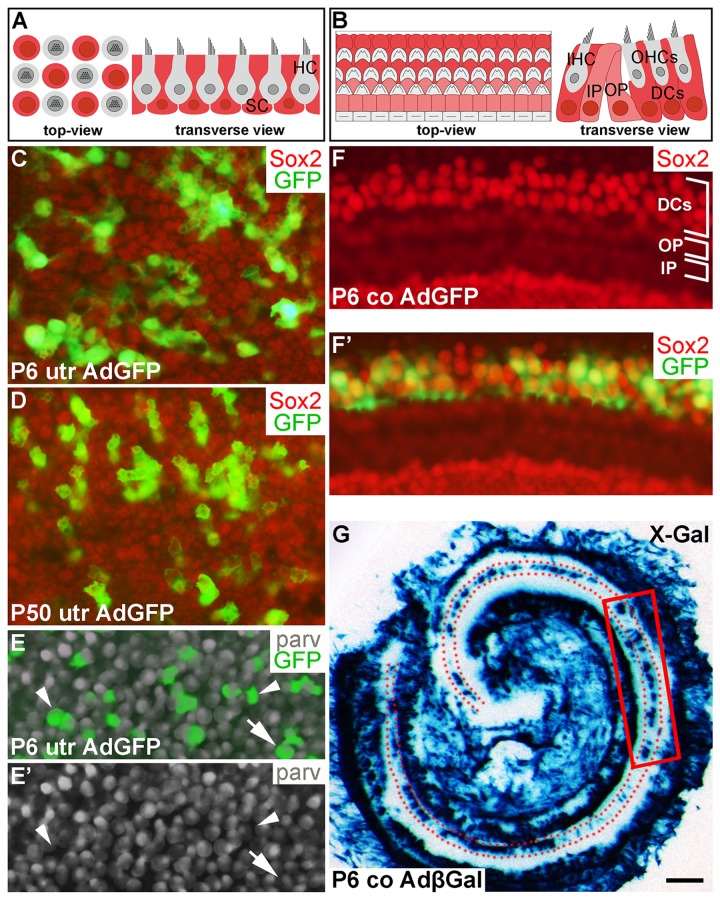
Adenoviruses transduce inner ear supporting cells in explant cultures. AdGFP- and AdβGal-infected utricles and cochleas analyzed after 3 DIV. (**A,B**) Schematic representation of the utricular (**A**) and cochlear (**B**) sensory epithelium, viewed from above (whole mount specimens) and in transverse plane. Utricular hair cells with the apical stereociliary bundle (grey) are located on top of a layer SCs (red). The cochlear sensory epithelium consists of one row of inner hair cells and three rows of outer hair cells (grey). Deiters' cells (red) are located underneath outer hair cells. Inner and outer pillar cells (pink) are positioned between the inner and outer hair cell rows. (**C,D**) AdGFP-infected P6 and P50 utricles double-labeled for GFP and Sox2 show transduction in SCs. The views are focused to the level of Sox2+ SC nuclei. (**E,E'**) In AdGFP-infected P6 utricle, a small part of parvalbumin+ hair cells are transduced (arrow), in addition to SCs (arrowheads). (**F,F'**) In P6 cochlea, Deiters' cells show AdGFP transduction, as opposed to the adjacent outer and inner pillar cells. (**G**) X-Gal histochemical staining shows a patchy pattern of AdβGal transduction in the area of Deiters' cells (dotted) along the length of the cochlear duct. The boxed area represents the region used for analysis. Abbreviations: utr, utricle; co, cochlea; AdβGal, adenovirus encoding β-galactosidase; AdGFP, adenovirus encoding green fluorescent protein; parv, parvalbumin; DCs, Deiters' cells; IP, inner pillar cell; OP, outer pillar cell; IHC, inner hair cell; OHCs, outer hair cells. Scale bar, shown in G: C-F', 20 µm; G, 180 µm.

In the present study, the sensory epithelia from postnatal day 6 (P6) utricles and cochleas, and from P50 utricles were prepared for organotypic cultures, as previously described [4]. The P6 and P50 SCs were used to represent the juvenile and adult SCs, respectively. Explants were maintained for 2 to 14 days *in vitro* (DIV). The auditory sensory epithelium, the organ of Corti, was not studied at adulthood due to difficulties in preserving *in vitro* the normal cytoarchitecture of the mature organ and the survival of its hair cells. SCs were marked by antibodies against Sox9 and Sox2 [4, 17]. In postnatal utricles, Sox2 is expressed in both SCs and hair cells. However, the nuclei of two cell types are located at different heights in the sensory epithelium and have different morphology, allowing cell type-specific analysis in whole mount surface preparations (Fig. 1A,B). In some experiments, hair cell-specific markers, parvalbumin and myosin 6 (myo6), were used.

Our previous work has established optimal conditions for transduction by adenoviruses encoding cD1 (AdcD1) and β-galactosidase (AdβGal) in adult utricular explants [4]. In the present study, also AdGFP reporter viruses were used to investigate viral tropism, an important issue, because our model organ comprises different cell types and because we studied different ages. AdGFP viruses transduced P6 and P50 utricular SCs, as detected by the presence of GFP+/Sox2+ (Fig. 1C,D) and GFP+/Sox9+ cells (data not shown) at 3 DIV. Transduction efficiency varied between individual explants, ranging from 20 to 50%. Only occasional AdGFP-infected hair cells were found in adult utricles (data not shown). P6 utricles showed higher amount of infected hair cells, based on quantification of parvalbumin+/GFP+ cells. The average infection rate of hair cells was 10% (10.1 ± 0.7, *n* = 3, total number of hair cells counted = 843). Together, even though infected hair cells were present in juvenile utricles, their amount was clearly outnumbered by infected SCs (Fig. 1E,E') [18].

In AdGFP- or AdβGal-infected P6 cochleas analyzed at 3 DIV, transgenes expressions were concentrated to Deiters' cells, a specific subtype of auditory SCs (Fig. 1F,F'). This expression was concentrated to the upper half of the cochlear duct, transduced Deiters' cells being often arranged in small patches (Fig. 1F',G). Hair cells were not transduced, based on the absence of GFP+/parvalbumin+ cells (data not shown). In the AdβGal-infected P6 cochlea shown in Fig. 1G, the boxed area represents the cochlear region analyzed in the present study. Taken together, under the experimental conditions used, the adenoviral serotype 5 vector (Ad5) with the *CMV* promoter preferentially transduces SCs in the juvenile and adult inner ear sensory epithelia, with an interesting Deiters´ cell-specific pattern in the cochlea.

### Response of juvenile and adult utricular supporting cells to AdcD1 infection

**Figure 2:**
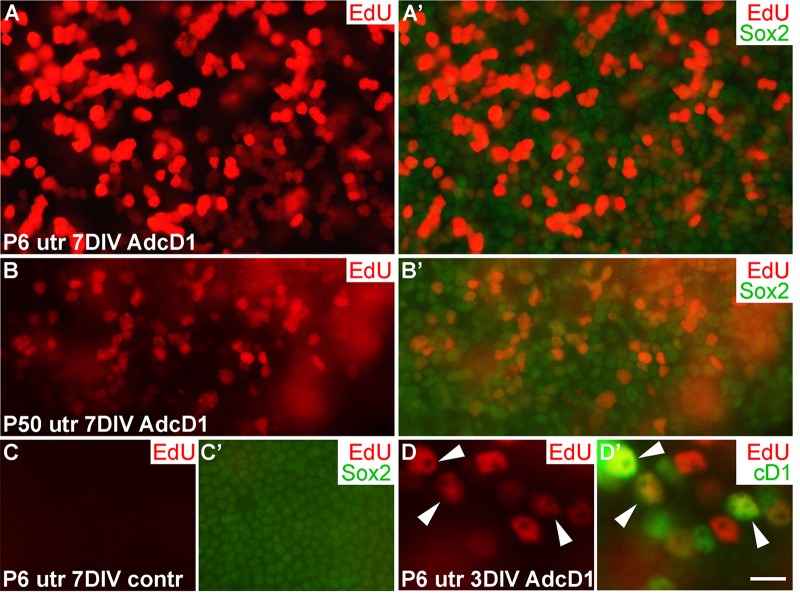
Proliferative response of juvenile and adult utricular supporting cells to cyclin D1 overexpression. Explants were pulsed with EdU for 24 h between days 2 and 3, and analyzed at 3 and 7 DIV. (**A,A'**) AdcD1-infected P6 utricle displays high numbers of EdU+/Sox2+ SCs. (**B,B'**) AdcD1-infected P50 utricle shows lower numbers of EdU+ SCs. (**C,C'**) Non-infected utricular explant is devoid of proliferating SCs. (**D,D'**) The AdcD1-infected P6 utricle shows EdU+/cD1+ SCs (arrowheads). In addition, there are only EdU-positive and only cD1-positive SCs due to cell cycle dynamics and EdU pulsing (see Results). Abbreviations: AdcD1, adenovirus encoding cyclin D1; utr, utricle. Scale bar, shown in D': A-C', 20 µm; D,D', 8 µm.

We used ectopic cD1 expression as a tool to force SCs into the cell cycle, based on the fact that many proliferation-promoting signaling pathways target this core cell cycle component. Particularly, cD1 is a central mediator of the proliferative response following activation of the Wnt/β-catenin pathway. It has been shown in mutant mouse models that Wnt/β-catenin activation increases proliferative activity of neonatal inner ear SCs [19]. To study cell cycle activity of SCs transduced by AdcD1, we pulsed explants with the thymidine analogue EdU that incorporates into replicating DNA, for 24 h between days 2 and 3. EdU labeling revealed high proliferative activity in AdcD1-infected P6 utricles at 7 DIV. Numerous clumps of small-sized, EdU+/Sox2+ nuclei of SCs were seen (Fig. 2A,A'), suggesting complete cell cycles. In AdcD1-infected P50 utricles, the amount of EdU+ SCs was clearly smaller, and pairs or clumps of EdU+ SCs were infrequent (Fig. 2B,B'). Combined with the prior findings of G2/M arrest of a large part of cell cycle reactivated adults SCs [4], these results point to inefficient expansion of the adult SC population. Non-infected and reporter virus-infected P6 and P50 explants were devoid of EdU+ SCs (Fig. 2C,C'; data not shown). Quantification revealed that on the average 20% (19.7 ± 2.4, *n* = 3, total number of SCs counted = 1194) of SCs were EdU-labeled in AdcD1-infected adult utricles at 3 DIV. In P6 utricles, this value was 50% (50.1 ± 2.2, *n* = 5, total number of SCs counted = 3631), reflecting the robust cell cycle activity of this juvenile cell population. Both P6 and adult utricles showed EdU-labeled SCs with cD1 expression, linking ectopic proliferation with adenovirus-mediated cD1 overexpression (Fig. 2D,D'; data not shown). Of note, because cD1 expression is rapidly downregulated in cells that initiate replication and because we retrospectively analyzed EdU-pulsed cells, SCs did not always show colabeling of cD1 and EdU.

### AdcD1-infected utricular supporting cells show a DNA damage response

**Figure 3:**
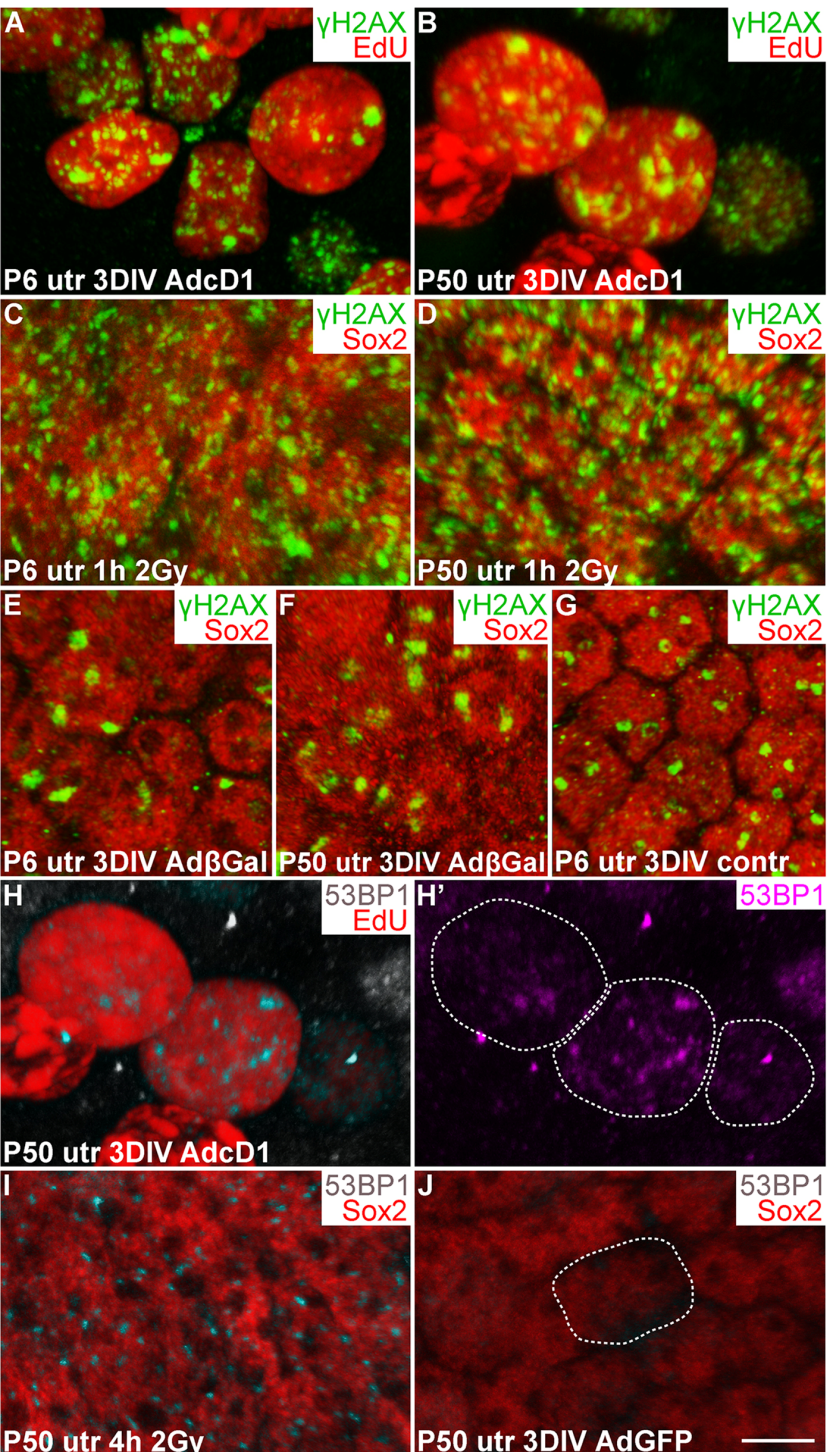
Utricular supporting cells show γH2AX and 53BP1 foci upon forced cell cycle re-entry, as revealed by confocal imaging. (**A,B**) At 3 DIV, AdcD1-infected P6 and P50 utricles display EdU+ SCs with numerous, small γH2AX foci. (**C,D**) Similar γH2AX profiles are seen 1 h post-irradiation in Sox2+ utricular SCs of both ages. (**E-G**) At 3 DIV, Sox2+ SCs in both AdβGal-infected P6 and P50 utricles, and in non-infected P6 utricle show one or two large γH2AX foci per nucleus. (**H,H'**) At 3 DIV, EdU+ SCs in AdcD1-infected P50 utricle display 53BP1 foci. Edu+ SC nuclei containing 53BP1 foci are outlined (**H'**). (**I**) Four hours post-irradiation, P50 utricular Sox2+ SCs show 53BP1 foci. (**J**) At 3 DIV, AdGFP-infected P50 utricle is devoid of 53BP1 foci. A Sox2+/GFP+/53BP1- nucleus is outlined. Abbreviations: AdcD1, adenovirus encoding cyclin D1; AdβGal, adenovirus encoding β-galactosidase; AdGFP, adenovirus encoding green fluorescent protein; Gy, gray; γH2AX, Ser 139 phosphorylated histone H2AX; utr, utricle. Scale bar, shown in J: A-J, 5 µm.

Oncogene-induced aberrant proliferation stimulates replication stress that can lead to DSB formation and DDR activation [20, 21]. We hypothesized that this is the case also with SCs following ectopic expression of the cD1 proto-oncogene. Furthermore, unresolved DSBs or other type of DNA damage might explain the low-level cell cycle activity of AdcD1-infected adult SCs compared to juvenile SCs (Fig. 2) [4]. To find out whether DSBs are formed in SCs, we compared using confocal microscopy the expression pattern of γH2AX, a DNA damage marker, in SCs challenged with AdcD1 and ionizing radiation (IR). γH2AX marks various forms of DNA damage, but the characteristic foci-like expression pattern seen in non-replicating cells following IR exposure represents DSBs [22]. Therefore, we used the γH2AX expression pattern seen 1 h after 2 gray (Gy) dose of IR as an established template of DSB profiles. Comparative analysis after 3 DIV showed that, similar to IR-treated SCs, AdcD1-infected and EdU-labeled (pulse between culture days 2 and 3) SCs of both ages expressed γH2AX as abundant small nuclear foci (Fig. 3A-D). These findings suggest that DSBs are induced in juvenile and adult SCs upon cell cycle re-entry.

To determine whether culture conditions and adenoviral transduction itself trigger DNA damage, non-infected as well as AdβGal- and AdGFP-infected utricles were analyzed after 3 DIV. One or two large γH2AX foci were found in SCs of both non-infected and reporter virus-infected utricles of both ages (Fig. 3E-G; data not shown). Thus, *in vitro* conditions induce low-level DNA damage, and viral transduction does not markedly alter this basal pattern. However, the DNA damage profile in these control explants is clearly different from the DSB-like profile with abundant small γH2AX foci seen following AdcD1-infection and IR. To confirm that the baseline γH2AX expression arising *in vitro* does not negatively affect proliferation, we prepared utricular cultures at P2, at the stage when a part of utricular SCs still naturally proliferates *in vivo* [16]. Both non-infected and AdGFP-infected P2 cultures showed EdU-labeled SCs after a 3-day-long pulsing period (data not shown), suggesting that the baseline γH2AX expression in explant cultures does not prevent natural SC proliferation.

As an additional marker for activated DDR, we used p53 binding protein 1 (53BP1), a component of the DDR pathway that localizes to the area of DSBs [23]. Similar to IR-challenged SCs, EdU-labeled SCs in AdcD1-infected utricles of both ages displayed 53BP1 foci (3H-I; data not shown). These foci were not detected in Sox2+/GFP+ SCs in explants infected with reporter viruses (Fig. 3J). We conclude that DDR is activated in juvenile and adult SCs upon forced cell cycle re-entry, with DSBs as a major type of DNA lesion.

### γH2AX foci resolution in AdcD1-infected utricular supporting cells

**Figure 4:**
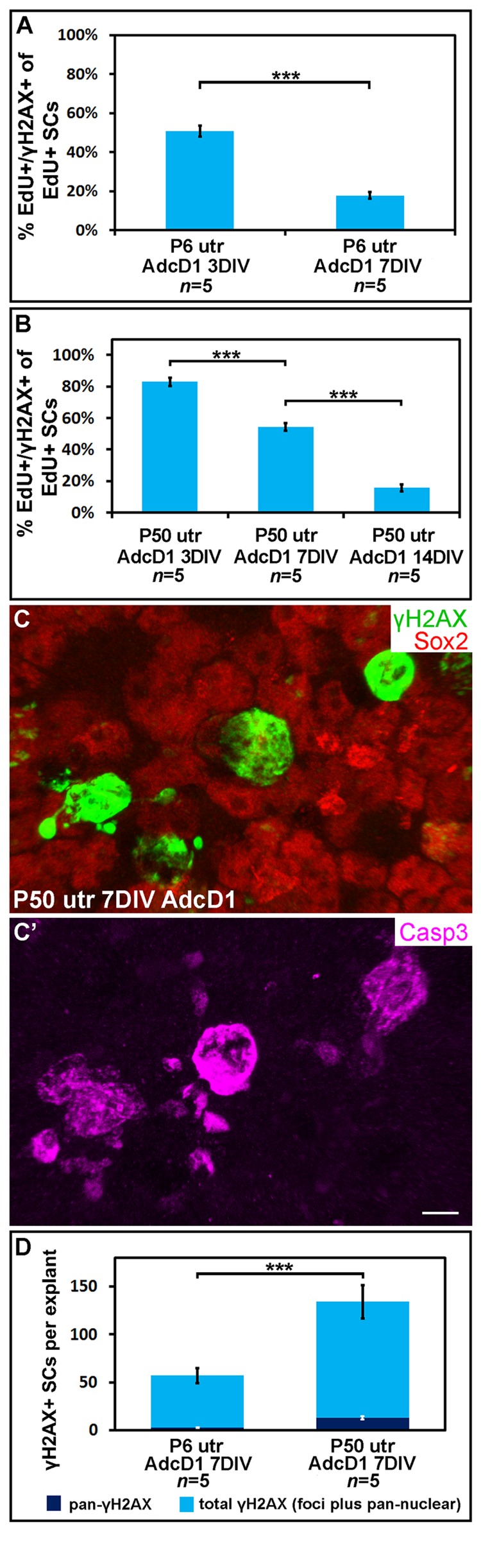
Utricular supporting cells show age-dependent resolution of γH2AX foci following cell cycle re-entry. AdcD1-infected P6 and P50 utricular explants were analyzed at 3, 7 and 14 DIV. (**A**) Quantification shows a prominent decrease in the amount of EdU+ SCs with γH2AX foci in P6 utricles from 3 to 7 DIV. **(B**) Quantification shows a prominent decrease in the amount of EdU+ SCs with γH2AX foci in P50 utricles not before 14 DIV. (**C,C'**) At 7 DIV, AdcD1-infected P50 utricles show SCs with intense, pan-nuclear γH2AX staining. These cells co-express cleaved caspase-3. (**D**) At 7 DIV, quantification shows a highly significant difference between AdcD1-infected juvenile and adult utricles in the proportion of γH2AX+ SCs with intense, pan-nuclear expression out of the total population of γH2AX+ SCs. Mean ± SEM and the number of explants (*n*) are shown. Statistical significance: ***, *p* < 0.005. Abbreviations: AdcD1, adenovirus encoding cyclin D1; Casp3, cleaved caspase-3; γH2AX, Ser 139 phosphorylated histone H2AX; utr, utricle. Scale bar, shown in C': C,C', 5 µm.

We next examined the temporal pattern of γH2AX expression in AdcD1-infected and EdU-labeled SCs (Fig. 4). In P6 utricles, the first replicating SCs were found at 2 DIV (EdU pulse between days 1 and 2). These cells showed γH2AX foci (data not shown), but the intensity of this expression was not as prominent as one day later when widespread γH2AX expression was found in the SC population, concomitantly with the high proliferative activity of these cells. Based on quantification at 3 DIV, 51% of EdU+ SCs (EdU pulse between culture days 2 and 3) showed the typical DSB pattern of γH2AX (SC numbers counted per explant: EdU+ 326.8 ± 27.8; γH2AX+/EdU+ 164 ± 12.2). This value was only 18% at 7 DIV (SC numbers counted per explant: EdU+ 274.8 ± 38.3; γH2AX+/EdU+ 48.6 ± 8.6) (Fig. 4A). The disappearance of SCs with γH2AX foci was not caused by cell death, based on the findings of only rare cleaved caspase-3-positive profiles in the juvenile explants analyzed at 7 DIV (data not shown). Of note, it is likely that the rather low percentage of EdU+ SCs with γH2AX foci at 3 DIV is an underestimate, because a part of the foci had already been resolved by this time point. These data on γH2AX resolution in AdcD1-infected juvenile SCs suggested successful DNA repair.

Next, using the same EdU pulsing regimen, the temporal pattern of γH2AX expression was analyzed in AdcD1-infected adult utricles (Fig. 4B). At 3 DIV, 83% of EdU+ SCs displayed γH2AX foci (SC numbers counted per explant: EdU+ 68.6 ± 4.1; γH2AX+/EdU+ 56.6 ± 2.4). This value was 54% at 7 DIV (SC numbers counted per explant: EdU+ 40.6 ± 10.8; γH2AX+/EdU+ 22.6 ± 6.5). By 14 DIV, the percentage of double-positive cells had decreased to 16% (SC numbers counted per explant: EdU+ 39.5 ± 3.7; γH2AX+/EdU+ 6 ± 0.4), a value comparable to that seen in juvenile utricles at 7 DIV (Fig. 4B). Compared to the juvenile specimens, these results showed that the γH2AX foci resolution is prolonged in adult utricles and, thus, suggested slower DNA repair.

At 7 DIV, in addition to EdU+ SCs with γH2AX foci, AdcD1-infected adult utricles displayed EdU+ SCs with intensive, pan-nuclear γH2AX staining (Figs. 3B, 4C). This γH2AX pattern has been previously associated with severe DNA damage and cell death [22]. Consistently, the majority of SCs with pan-nuclear γH2AX co-expressed cleaved caspase-3, a marker for apoptotic death (Fig. 4C,C'). Quantification at 7 DIV showed a highly significant difference (*p* < 0.005) between the relative amount of γH2AX+ SCs with intensive, pan-nuclear staining in P6 and P50 utricles (Fig. 4D). This γH2AX expression profile was absent in explants transduced by reporter viruses (data not shown). Together, these data suggest that prolonged DNA damage resolution in cell cycle reactivated adult SCs predisposes these cells to apoptosis.

### The repair protein Rad51 is dynamically expressed in AdcD1-infected utricular supporting cells

Rad51 is a DNA repair protein engaged in homologous recombination repair (HRR) and is detected in the respective repair foci. HRR is relevant in the context of cell cycle reactivated SCs investigated in the present work, because it predominates in S and G2 phases of the cell cycle [24]. We used Rad51 as a marker for HRR, and examined whether its expression correlate with the observed γH2AX foci resolution.

**Figure 5:**
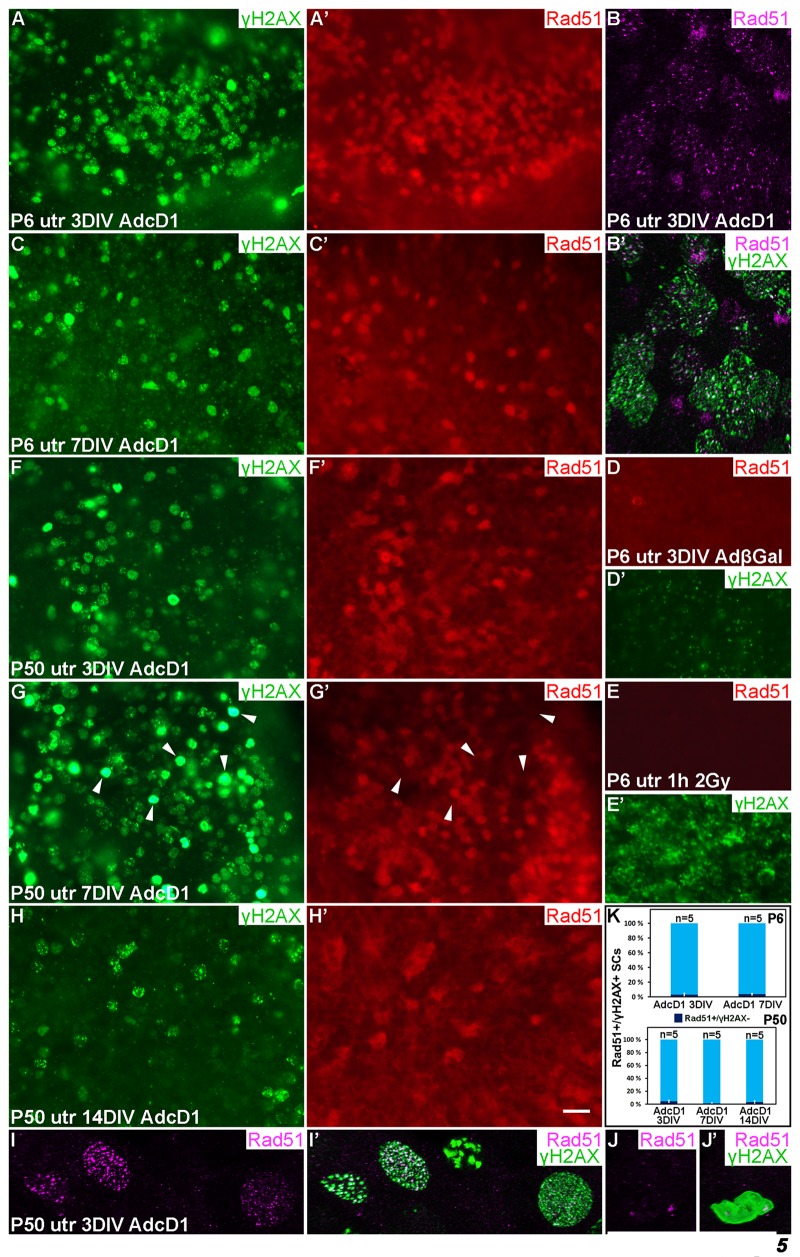
Dynamics of the DNA repair protein Rad51 in cell cycle reactivated supporting cells. Adenovirus-infected utricular explants were maintained for 3 to 14 DIV and double-labeled for γH2AX and Rad51. Imaging by conventional (**A,A',C-H'**) and confocal microscopy (**B,B',I-J'**). (**A,A'**) At 3 DIV, AdcD1-infected P6 utricle shows Rad51 upregulation in SCs with γH2AX foci. (**B,B'**) High magnification view of an AdcD1-infected P6 utricle showing Rad51 expression as small foci that partially colocalize with the γH2AX foci. (**C,C'**) By 7 DIV, the reduction in Rad51 levels is paralleled by γH2AX resolution. (**D,D'**) AdβGal-infected P6 utricle lacks Rad51 expression. (**E,E'**) P6 utricle exposed to ionizing radiation and analyzed 1 h post-irradiation contains SCs with DSB-like γH2AX foci. These cells lack Rad51 expression. (**F,F'**) At 3 DIV, also the AdcD1-infected P50 utricle shows Rad51 induction in γH2AX+ SCs. (**G,G'**) Rad51 expression is maintained at 7 DIV. Note that adult SCs with bright, pan-nuclear γH2AX staining lack or show only weak Rad51 expression (arrowheads). (**H,H'**) Rad51 levels are reduced in the adult utricle by 14 DIV, paralleling γH2AX resolution. (**I,I'**) High magnification view shows Rad51 foci in AdcD1-infected adult SCs and that these foci partially colocalize with γH2AX foci. (**J,J´**) High magnification view shows that adult SCs with pan-nuclear γH2AX staining express only low levels of Rad51. (**K**) Quantification shows that, at all timepoints studied and both in P6 and P50 utricles, Rad51 expression closely parallels that of γH2AX. Only 3-5% of Rad51+ SCs were negative for γH2AX. Mean ± SEM and the number of explants (*n*) are shown. Abbreviations: AdcD1, adenovirus encoding cyclin D1; AdβGal, adenovirus encoding β-galactosidase; γH2AX, Ser 139 phosphorylated histone H2AX; utr, utricle. Scale bar, shown in H': A,A',C-H', 20 µm; B,B',I-J', 5 µm.

At 3 DIV, Rad51 was upregulated in AdcD1-infected P6 utricles, specifically in SCs with γH2AX foci (Fig. 5A,A'). This was readily seen under light microcopy and low magnification. The nuclear Rad51 expression appeared fuzzy at low magnification, because the expression was formed by numerous small foci, as revealed by confocal imaging at high magnification (Fig. 5B,B'). Interestingly, in juvenile SCs, Rad51 expression was downregulated by 7 DIV, paralleling the resolution of γH2AX foci (Fig. 5C,C'). AdβGal-infected explants did not show Rad51 expression (Fig. 5D,D'). As an additional control, IR-challenged, non-proliferating SCs lacked Rad51 expression (Fig. 5E,E'), consistent with the predominance of non-homologous end-joining rather than HRR in DSB repair in non-proliferating cells. Together, in juvenile SCs, HRR was induced upon ectopic proliferation and DDR activation, and was down-regulated concomitantly with successful DNA repair.

Also AdcD1-infected adult utricles showed Rad51 induction at 3 DIV, but as opposed to juvenile explants, downregulation was not observed before 14 DIV. This is consistent with the resolution of γH2AX foci by this late post-infection time point (Fig. 5F-H'). Similar to younger SCs, confocal microscopy revealed Rad51 expression as small nuclear foci in adult SCs (Fig. 5I,I'). Quantification showed that Rad51 expression closely paralled that of γH2AX both in juvenile and adult SCs (Fig. 5K). As the presence of γH2AX foci matched with EdU labeling (Fig. 4A,B), we conclude that Rad51+ cells represent cell cycle reactivated and DNA damaged SCs. Interestingly, at 7 DIV, SCs with the strong, pan-nuclear γH2AX expression lacked or showed only very weak Rad51 expression (Fig. 5G,G',J,J'). Because many of these cells were also positive for cleaved caspase-3 (Fig. 4C,C'), these data link delayed DNA repair to cell death.

### AdcD1-triggered cell cycle re-entry of auditory supporting cells

**Figure 6:**
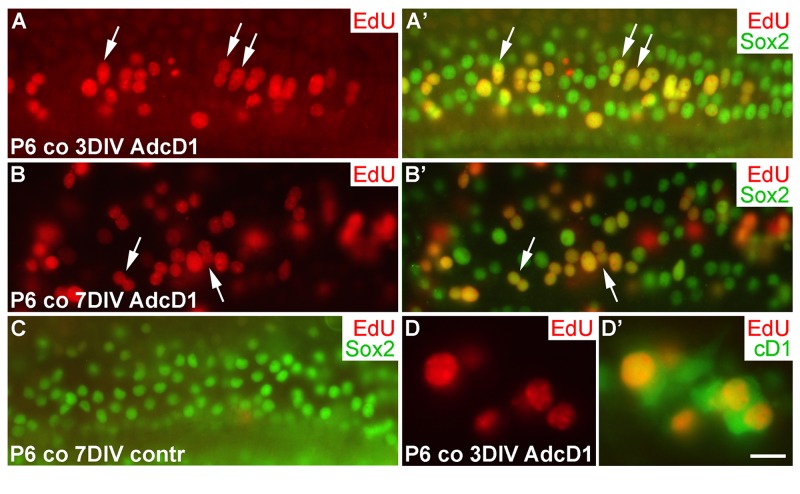
Proliferative response of juvenile cochlear supporting cells to cyclin D1 overexpression. (**A,A'**) The AdcD1-infected cochlea shows EdU+/Sox2+ Deiters' cells at 3 DIV. (**B,B'**) At 7 DIV, despite cell cycle re-entry of Deiters' cells, this cell population lacks clear signs of supernumerary cells. (**C**) A non-infected cochlear explant is devoid of proliferating SCs. (**D,D'**) An AdcD1-infected P6 cochlea shows EdU+/cD1+ SCs. Abbreviations: co, cochlea; AdcD1, adenovirus encoding cyclin D1. Scale bar, shown in D': A-C, 20 µm; D,D', 10 µm.

We next studied cell cycle activity in cochlear SCs transduced by AdcD1 at P6. Consistent with the transduction pattern revealed by reporter viruses (Fig. 1F',G), the upper half of AdcD1-infected cochlear explants showed EdU labeling exclusively in Sox2+ Deiters' cells (EdU pulse between days 2 and 3). These double-positive cells were found both at 3 and 7 DIV (Fig. 6A-B'). Non-infected and reporter virus-infected cochleas were devoid of EdU+ cells (Fig.6C; data not shown). Following AdcD1 infection, similar to utricles, cochleas showed EdU-labeled Deiters' cells with cD1 upregulation (Fig. 6D,D').

**Figure 7:**
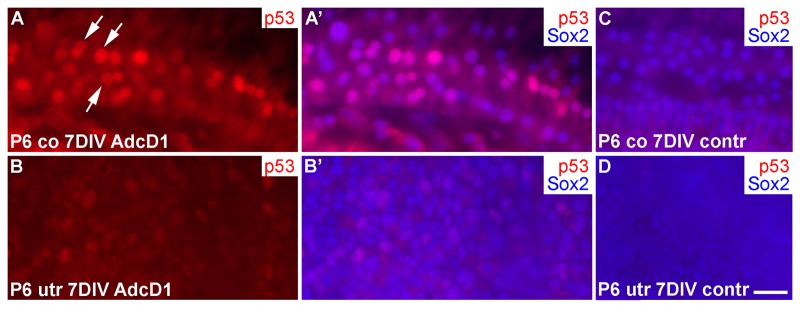
p53 induction in SCs of cochlear explants transduced by AdcD1. (**A-B'**) At 7 DIV, p53 is upregulated in Sox2+ Deiters' cells of P6 cochleas, especially in nuclear doublets and in the large, likely replicating nuclei (arrows). Age-matched utricles show weak or undetectable p53 upregulation. (**C,D**) In non-infected explants, p53 expression is undetectable. Abbreviations: co, cochlea; AdcD1, adenovirus encoding cyclin D1; utr, utricle. Scale bar, shown in D: A-D, 20 µm.

Several EdU+ doublets of Deiters' cells were found in AdcD1-infected P6 cochleas. In many cases, the nuclei of these doublets appeared to be firmly connected with each other, suggesting for incomplete cytokinesis (Fig. 6A-B'). Consistently, the Deiters' cell population did not show clear signs of expansion, based on morphological examination and comparison to the control (non-infected and reporter virus-infected) cochleas at 7 DIV (Fig. 6A-C). An underlying reason might be the induction of p53, an anti-proliferative transcription factor often activated upon unscheduled proliferation [25]. Indeed, we found p53 upregulation in Deiters' cells of AdcD1-infected cochleas, particularly in cell doublets, but also in cells with large nuclei, typical to replicating cells (Fig. 7A,A**'**). Interestingly, SCs of age-matched, AdcD1-infected utricles did not show p53 upregulation (Fig. 7B,B**'**), consistent with their high proliferative activity (Fig. 2A,A'). p53 expression was undetectable in control explants of both the cochlea and utricle (Fig. 7C,D).

Thus, these data suggested an inverse correlation between the proliferative activity and p53 response in the cochlear Deiters' cells.

### γH2AX foci accumulate in cell cycle reactivated auditory supporting cells *in vitro* and *in vivo*

**Figure 8:**
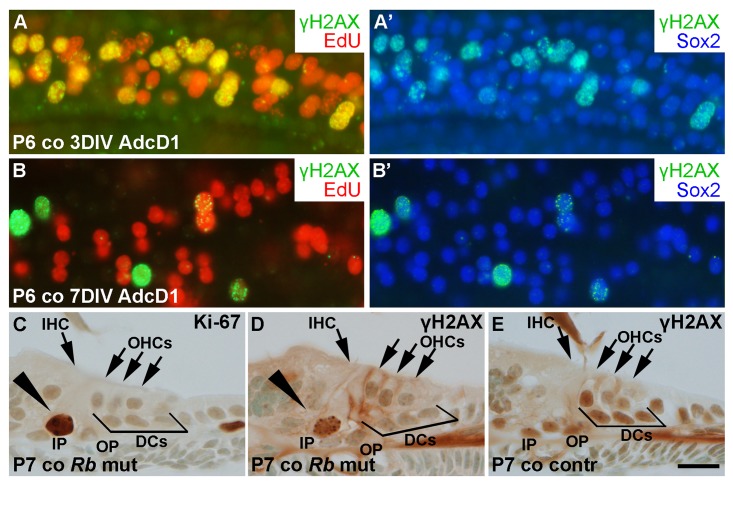
Unscheduled proliferation is a specific trigger for the DNA damage response in auditory SCs *in vitro* and *in vivo*. (**A,A'**) AdcD1-infected P6 cochleas pulsed with EdU (for 24 h between days 2 and 3) and maintained for 3 DIV show accumulation of γH2AX foci in cell cycle reactivated EdU+ Deiters' cells. (**B,B'**) Most EdU+ Deiters' cells show γH2AX downregulation by 7 DIV. (**C-E**) Cross-sections through the cochlea of a *Rb^loxP/loxP^;Fgfr3-iCre-ER^T2^* mutant mouse at P7 show Ki-67-stained pillar cells (arrowhead in C). γH2AX foci can be seen in pillar cells (arrowhead in D) of mutant, but not control mice. Abbreviations: AdcD1, adenovirus encoding cyclin D1; γH2AX, Ser 139 phosphorylated histone H2AX; co, cochlea; DCs, Deiters' cells; IHC, inner hair cell; OHCs, outer hair cells; IP, inner pillar cell; OP, outer pillar cell; *Rb* mut, *Rb^loxP/loxP^;Fgfr3-iCre-ER^T2^*. Scale bar, shown in E: A-E, 20 µm.

We next studied the dynamics of γH2AX expression in AdcD1-infected Deiters' cells. At 3 DIV, P6 cochlear explants showed EdU+/Sox2+ Deiters' cells with γH2AX foci, typical to DSBs (Fig. 8A,A'). At 7 DIV, most of these cells, including most of the cell doublets, lacked γH2AX foci (Fig. 8B,B'). Combining these results to the utricular data, we conclude that the dynamics of DSB resolution in the inner ear SCs is age-dependent.

To reveal whether the accumulation of γH2AX foci is a specific response to cD1 overexpression or whether it is a general response to cell cycle re-entry, the *retinoblastoma* (*Rb*) gene was inactivated *in vivo* in auditory SCs. We generated *Rb^loxP/loxP^;Fgfr3-iCre-ER^T2^* mutant mice and injected the pups with tamoxifen in order to inactivate *Rb* in auditory SCs from P2 onward. The *Fgfr3-iCre-ER^T2^* transgenic mouse line mediates efficient and cell type-specific recombination in the cochlea, as previously shown [26]. Cross-sections were prepared from the cochleas of mutant animals and littermate controls at P7, and stained for γH2AX and Ki-67. Pillar cells, a subtype of auditory SCs that similar to Deiters' cells do not proliferate postnatally, showed unscheduled proliferation in the mutant mice during the first two postnatal weeks (Fig. 8C). We focused our analysis on pillar cells, because only a few Deiters' cells of the *Rb^loxP/loxP^;Fgfr3-iCre-ER^T2^* mice showed cell cycle reactivation, consistent with prior data [27]. In contrast to control specimens, pillar cells of mutant animals showed induction of γH2AX foci (Fig. 8D,E). Thus, these data show that both ectopic cD1 *in vitro* and genetic inactivation of a negative cell cycle regulator *in vivo* can trigger cell cycle re-entry of auditory SCs and that this event is linked with DSB accumulation.

### Supporting cell-to-hair-cell transdifferentiation does not trigger DNA damage

**Figure 9:**
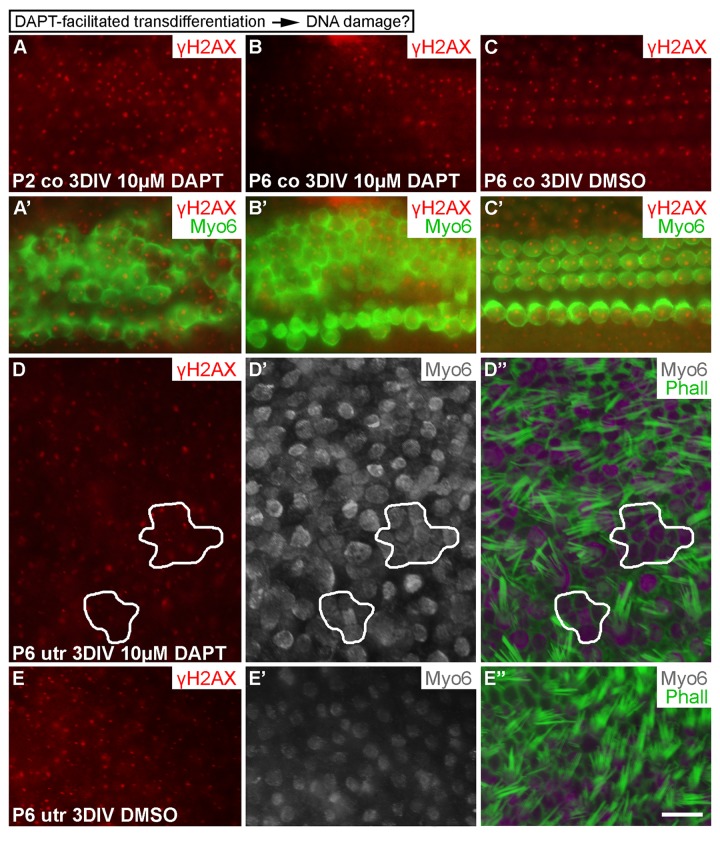
Transdifferentiation does not trigger DNA damage. (**A-C'**) In both P2 and P6 cochlear explants, DAPT treatment for 3 DIV triggers production of ectopic, myo6-labeled outer hair cells. The extent of γH2AX expression is similar as in non-treated cochleas. (**D-E''**) Similarly, in P6 utricular explants, DAPT treatment for 3 DIV triggers production of ectopic, myo6-labeled hair cells. New hair cells (outlined in D-D'') are in direct contact with each other and lack mature stereociliary bundles, based on myo6 and phalloidin labelings. The extent of γH2AX expression in ectopic hair cells is similar to normally developed hair cells and to hair cells in vehicle-treated control utricles. Abbreviations: co, cochlea; γH2AX, Ser 139 phospho-rylated histone H2AX; Myo6, myosin 6; Phall, phalloidin; utr, utricle. Scale bar, shown in E'': A-C', 20 µm; D-E'', 13 µm.

Having found that DSBs are formed in cell cycle reactivated SCs and that DNA damage resolution directs proliferative activity, we investigated whether SC-to-hair-cell transdifferentiation, the other main step in the regeneration process, triggers DNA damage as well. Transdifferentiation can be triggered by the γ-secretase inhibitor DAPT that inactivates Notch signaling [28, 29]. In addition to P6 explants, we treated P2 cochlear explants with DAPT, because SC's response to DAPT is more pronounced at this earlier age. Cochleas were analyzed after a 3-day-long DAPT incubation. It is known that DAPT stimulates new hair cell formation during this whole culturing period [30]. Unlike control specimens (treated with DMSO), DAPT-treated cochleas showed supernumerary, myo6-positive hair cells, based on surface views where the normal mosaic-like organization of the sensory epithelium (Fig. 1B) was in places lost. In these areas, hair cells were in contact with each other, unlike in control explants where hair cells were contacted by SCs. Notably, these ectopic hair cells did not show γH2AX induction above the level seen in normal hair cells of the same explants or of control cultures (Fig. 9A-C'). Similar results were obtained in P6 and adult utricles after a 3-day-long DAPT treatment. Regions were found in these specimens where myo6-positive hair cells lacked mature stereociliary bundles, based on phalloidin staining that marks filamentous actin-rich stereocilia, suggesting that these cells had been generated through trans-differentiation (Fig. 9D-E''; data not shown). Thus, as opposed to forced cell cycle re-entry, stimulation of transdifferentiation of SCs does not trigger DNA damage.

### Uncoupling between forced proliferation and transdifferentiation of utricular supporting cells

**Figure 10:**
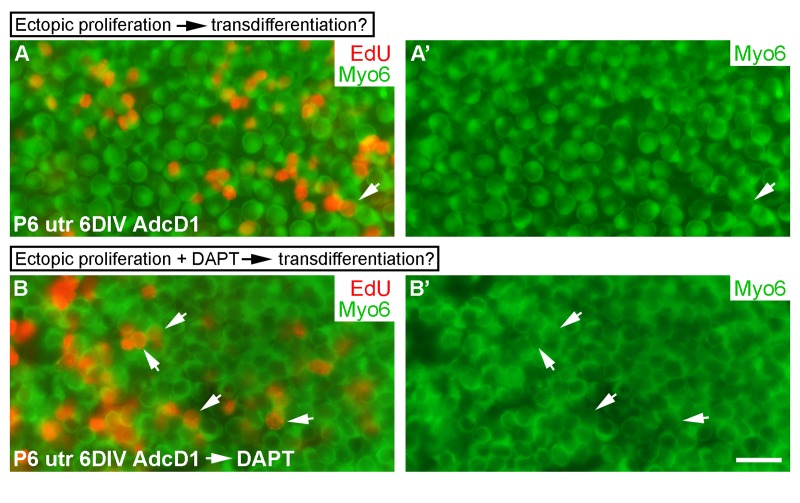
Uncoupling between forced proliferation and transdifferentiation. (**A,A'**) AdcD1-infected P6 utricles show rare EdU+/myo6+ hair cells at 6 DIV (arrow). (**B,B'**) AdcD1-infected and DAPT-treated P6 utricles contain high numbers of EdU+/myo6+ hair cells (arrows) at 6 DIV (regimen of the treatments explained in Results). Abbreviations: AdcD1, adenovirus encoding cyclin D1; Myo6, myosin 6; utr, utricle. Scale bar, shown in B': A-B', 20 µm.

Next we wanted to investigate whether forced cell cycle re-entry is coupled to transdifferentiation, because natural regeneration in non-mammalian inner ears often involves SC divisions, followed by transdifferentiation of a part of the progeny into hair cells [1]. We used AdcD1-infected P6 utricles for these experiments, applying a 24-h-long EdU pulse between days 2 and 3. We took into account the fact that, in addition to SCs, a small part of hair cells of juvenile utricles are infected by adenoviruses (Fig. 1E,E'). At 6 DIV, the amount of EdU+/myo6+ hair cells was less than 10% in AdcD1-infected utricles, and this value corresponded to the amount of hair cells in the explants infected with reporter viruses (Figs. 1E,E' and 10A,A'). These results argue against coupling between forced proliferation and transdifferentiation of postmitotic SCs.

We then asked whether forced proliferation can be combined with DAPT-induced transdifferentiation. AdcD1-infected P6 utricles were cultured for 3 days (EdU pulse between days 2 and 3) and then treated with DAPT for another 3 days. After 6 DIV, a robust, threefold increase in EdU+/myo6+ hair cells was found in these specimens compared to only AdcD1-infected explants (AdcD1: number of myo6+ cells counted 768, number of EdU+/myo6+ cells 22, *n* = 3 explants; AdcD1 plus DAPT: corresponding values 1096 and 109, *n* = 3) (Fig. 10A-B'). Thus, preceding cell cycle reactivation does not prevent pharmaco-logical stimulation of SCs to transdifferentiate into hair cells.

## DISCUSSION

Current research puts efforts on understanding the mechanisms that restrict hair cell regeneration in the mammalian inner ear and how these restrictions could be overcome, the aim being the design of therapeutic strategies to replace lost hair cells by new ones. SCs serve as a potential platform for these regenerative interventions. We have studied SC's response to forced cell cycle re-entry and taken into account prior data on the decline of proliferative potential of these cells during the early postnatal maturation period [15]. Juvenile SCs are proliferation-proficient; they are mostly postmitotic [16], but their proliferation can be stimulated *in vitro* by exogenous mitogens, manipulation of cell cycle regulators and conditions that promote stemness [31]. These stimuli have only little effect on cell cycle activity of adult SCs. Although hair cell re-growth might be achieved through SC-to-hair-cell transdifferentiation, SC depletion at the expense of new hair cells may not be a viable therapeutic approach [32]. Unscheduled cell cycle re-entry and DNA damage have been linked to neurodegenerative diseases [33] and to restrictions in stem cell reprogramming [2]. Based on these considerations, understanding of DNA damage signaling in adult SCs is important for the development of safe and efficient strategies of proliferative regeneration, an event that could potentially be followed by transdifferentiation into hair cells.

Present results demonstrate that Ad5 viruses with the *CMV* promoter efficiently target postnatal SCs in explant cultures. In the juvenile cochlea containing different types of SCs, the transduction was specific to Deiters' cells. This SC subtype-specific infection and the fact that auditory hair cells were largely non-infected, make Ad5 vectors excellent tools in targeted manipulations of a specific population of the auditory sensory epithelial cells. Besides possessing regenerative potential, Deiters' cells are the key cellular players in the pathophysiologically important wound healing in the auditory sensory epithelium [34]. Thus, Ad5-mediated manipulations could be useful in revealing molecular regulation underlying these events. Prior studies have shown that adenoviral tropism changes rapidly during development: mainly hair cells are transduced at late-embryogenesis, while both hair cells and SCs are transduced around birth. Thereafter, during the early postnatal maturation period, the tropism gradually shifts towards SCs [4, 18, 35].

We found that γH2AX was expressed, in addition to reporter virus-transduced SCs, in SCs of non-infected explants. These results suggest that, rather than viral transduction, stressors such as oxidative stress known to be associated with *in vitro* conditions might cause γH2AX induction [36]. However, the intensity and pattern of this basal γH2AX expression was clearly different compared to the expression seen in AdcD1-infected SCs. Nuclei of these cells showed numerous small γH2AX and 53BP1 foci that characterize DSB damage. Thus, we conclude that AdcD1-induced DSBs are caused by the transgene, a core cell cycle component that promotes G1/S transition, rather than by adenoviral transduction itself. These data are supported by our *in vivo* results in mice with the conditional, inducible inactivation of *Rb*, a downstream target of the D-type cyclin/cyclin-dependent kinase 4/6 complexes. In these mutant animals, abnormal cell cycle re-entry of juvenile auditory supporting cells, mostly pillar cells, was coupled with the accumulation of γH2AX foci. Similarly, γH2AX induction has been demonstrated in *Rb*-depleted, cell cycle reactivated adult forebrain neurons *in vivo* [37]**.** Furthermore, our results showing that stimulation of SC-to-hair cell transdifferentiation by the γ-secretase inhibitor DAPT does not elicit DSB-like γH2AX foci speak for the cell cycle re-entry being a specific trigger for this DNA damage.

One of the key findings of the current study was the differences in the DDR and DNA repair dynamics between AdcD1-infected juvenile and adult SCs. The results showing rapid resolution of γH2AX foci in juvenile SCs indicated that successful DNA repair was accomplished. This was also suggested by the foci formation and subsequent resolution of the core HRR component Rad51, indicating the engagement of HRR. In contrast, the slower resolution of γH2AX and Rad51 foci in adult SCs implies a delayed DNA repair. Further, the findings of adult SCs with intense, pan-nuclear γH2AX that were positive for cleaved caspase-3 and showed only negligible Rad51 expression suggested that incomplete DNA repair could lead to apoptosis. These results are consistent with prior data showing signs of apoptosis of cell cycle reactivated SCs of the adult utricle upon c-Myc overexpression [38]. What might be the mechanisms underlying slower kinetics of DNA damage signaling in adult SCs? Cellular differentiation is accompanied by an increase in heterochromatinization that is known to affect DNA repair [39]. DSBs occurring near or within hetero-chromatin, the tightly packed and transcriptionally inactive form of DNA, are repaired more slowly due to the chromatin challenge [40]. Thus, an increase in heterochromatin accumulation and condensation along maturation might delay DNA repair and impair survival and cell cycle progression of adult SCs.

In response to AdcD1 transduction, in addition to differences between juvenile and adult utricular SCs, we found differences in proliferative activity between age-matched utricular and cochlear SCs, at the juvenile stage. At this stage, SCs of both organs are postmitotic, but are undergoing structural maturation. Production of ectopic Deiters' cells of the cochlea was less evident than that of utricular SCs. Specifically, the numerous doublets of EdU-labeled Deiters' cells and the signs of impaired separation of the nuclei of the doublets suggested that the cell cycles were incomplete. Interestingly, this has also been found in developing hair cells after cell cycle reactivation [11, 41]. We found that p53, a mediator of cycle arrest and apoptosis, was upregulated in Deiters' cells of AdcD1-infected cochkeas, in most cases in the cell doublets. p53 induction was not found in SCs of AdcD1-infected, juvenile utricles that showed robust cell cycle activity. Although these results link p53 upregulation with incomplete cell cycles of Deiters' cells, direct evidence of the role of p53 in these cells remains to be shown, similar to the understanding of the mechanisms behind p53 upregulation.

AdcD1-triggered proliferation of P6 utricular SCs was not followed by transdifferentiation into hair cells. Thus, upon forced proliferation of postmitotic SCs, the two regenerative events seem to be uncoupled. Recently, a pulse-labeling study with thymidine analogs in the mouse utricle *in vivo* showed that, at birth and a few days thereafter, a part of SCs or SC-like-cells still naturally proliferate and the progeny produced from those divisions differentiate into hair cells [16]. In another *in vivo* study in which hair cells were killed form the neonatal mouse utricle, SC's proliferative activity increased, resulting in mitotic hair cell replacement [42]. Combined with our results with slightly older SCs, the acquired postmitotic status, maintained by negative cell cycle regulators [31] and epigenetic mechanisms [43], is likely to underlie the observed uncoupling of forced cell cycle re-entry and transdifferentiation. Also, our findings showing that, in the postmitotic SCs, transdifferentiation can be pharmacologically stimulated after forced cell cycle re-entry, speak for independent molecular regulation of the two events.

To conclude, we have here used the inner ear SCs as models of postmitotic cells to understand the limitations in DNA damage signaling in the context of cell cycle reactivation. Several other types of quiescent cells, such as tissue-resident quiescent stem cells, are likely to pose similar barriers in the attempts to stimulate proliferative activity. Most studies on DNA damage signaling have been performed with cultured single cells. Our explant culture model retains many aspects of *in vivo* tissue integrity, yet it allows manipulation and analysis at the cellular level. It appears that the capacity of adult SCs to proliferate is a staggering step in the efforts to stimulate hair cell re-growth. Therefore, dedifferen-tiation of mature SCs towards immature-like cells may be required for successful proliferation. In addition to being a component of DDR signaling, histone H2A variant H2AX is an epigenetic regulator of proliferation, as recently shown in quiescent adult stem cells [44]. Thus, the link between DDR and epigenetic signaling might be an important factor in creating barriers against proliferative regeneration also in the inner ear SCs. Similar to the findings made in β-cells of the adult human pancreas [45], our results show that restrictions in DNA damage signaling form an important barrier of proliferation of adult SCs.

## METHODS

### Mice

Explant cultures of the utricular and cochlear sensory epithelia were prepared from NMRI mice, and histological sections from the inner ears of *Rb^loxP/loxP^;Fgfr3-iCre-ER^T2^* mice. Conditional, inducible inactivation of *Rb* in auditory SCs of these mutant mice was induced by tamoxifen at P2 and P3, as described previously [26]. *Rb^loxP/loxP^* and *Fgfr3-iCre-ER^T2^* mice were genotyped by PCR as described in the original publications [46, 47]. The day of birth was considered as P0. All animal work has been conducted according to relevant national and international guidelines.

### Explant cultures and viral infections

Explant cultures were established at P2 and P6 from the cochlea, and at P2, P6 and P50 from the utricle. Explants were maintained on pieces of Nuclepore filter membrane (Whatman) placed on a metal grid in Dulbecco's modified Eagle's DMEM/F-12 medium supplied with 2 mM L-glutamine and penicillin (100 U/mL) (Gibco/Invitrogen) and 10% fetal bovine serum (FBS) (HyClone/Thermo Scientific). Incubations were done in a humidified 5% CO_2_ atmosphere at 37°C. Ad5 vectors harbouring the *βGal*, *GFP* or *cD1* transgenes and *CMV* promoter were used. Cloning and propagation of the viruses have been described previously [48]. Cochlear and utricular explants were infected for 16 and 7 h, respectively, with Ad*β*Gal (4.2 × 10^7^ pfu/ml), AdGFP (3.6 × 10^7^ pfu/ml) or AdcD1 (2.9 × 10^7^ pfu/ml). Infections were done in 30 µl drops of medium containing 5% FBS. Thereafter, explants were maintained for 2 to 14 DIV in medium containing 10% FBS. Medium was changed every other day.

### Whole mount specimens

Explants were fixed with 4% paraformaldehyde (PFA) in phosphate buffered saline (PBS) for 10 min and permeabilized in PBS containing 0.25% Triton-X-100 (PBS-TX). Double- and triple-immunofluorescent stainings were performed in PBS-TX and 10% normal serum, using the following antibodies: goat parvalbumin (Swant, #PVG-214, 1:2000); rabbit cleaved caspase-3 (Asp-175) (Cell Signaling Technology, #9661, 1:250); rabbit Sox9 (Millipore, #AB5535, 1:2000); mouse γH2AX (Millipore, #05-636, clone JBW301, 1:500); goat Sox2 (Santa Cruz Biotechnology, #SC-17320, 1:1000); rabbit Rad51 (H-92) (Santa Cruz Biotechnology, #SC-8349, 1:800), rabbit GFP (Molecular Probes/Invitrogen, #A6455, 1:2000); rabbit p53 (Novocastra, #NCL-p53-CM5p, 1:500); rabbit myosin 6 [49]; rabbit 53BP1 (Cell Signaling Technology, #4937, 1:350). Following antibody incubations, filamentous actin-containing stereociliary bundles were visualized using Oregon Green-labeled phalloidin (Molecular Probes/Invitrogen, 1:400). Secondary antibodies conjugated to Alexa 350, 488, 568, 594 and 647 were used for visualization, and ProLong Gold anti-fade reagent (Molecular Probes/Invitrogen) for mounting. β-Galactosidase staining was performed by incubating fixed explants in X-gal staining solution [1 mg/ ml X-gal (5-bromo-4-chloro-3-indolyl-β-D-galactopyranoside; Promega), 5 mM K_3_Fe(CN)_6_, 5 mM K_4_Fe(CN)_6_, 2 mM MgCl_2_, 0.025% sodium deoxycholate and 0.025% Nonidet P-40] for 15 min at room temperature and mounted in ProLong Gold medium.

### EdU pulsing and labeling

Adenovirus-infected explants were given a single pulse of 10 µM EdU (5-ethynyl-2'-deoxyuridine; Molecular Probes/Invitrogen) for 24 h between days 2 and 3, and fixed at 3, 7 and 14 DIV. A part of infected explants were given a single pulse of EdU for 24 h between days 1 and 2, and fixed immediately thereafter, at 2 DIV. Non-infected P2 utricles were given a single pulse of EdU every 24 h for three consecutive days, followed by fixation at 3 DIV. Specimens were stained according to manufacturer's instructions using the Click-iT EdU Imaging Kit (Invitrogen) and mounted in ProLong Gold medium.

### Histological sections

Inner ears from *Rb^loxP/loxP^;Fgfr3-iCre-ER^T2^* mice were perilymphatically fixed with 4% PFA at P7, and immersed in the fixative overnight. Specimens were decalcified for 6 h in 0.5 M EDTA, pH 8.0, followed by embedding in paraffin and cutting to 5-μm-thick sections. Epitopes were unmasked by microwave heating (800 W) in 10 mM citrate buffer, pH 6.0, for 10 min. Sections were stained overnight with the γH2AX and rabbit monoclonal Ki-67 (LabVision/Thermo Scientific, #RM-9106, clone SP6, 1:250) antibodies in PBS-TX. Detection was done using the Mouse-on-Mouse (γH2AX) and Vectastain Elite ABC (Ki-67) kits and the DAB Detection kit (Vector Laboratories). Sections were counterstained with 3% methyl green and mounted in Permount (Fisher Scientific).

### DAPT treatment

Cochlear (P2 and P6) and utricular (P6 and P50) explants were treated with the γ-secretase inhibitor DAPT (N-[N-(3,5-Difluorophenacetyl)-L-alanyl]-S-phenylglycine t-butyl ester; Sigma Aldrich, #D5942) or the DMSO vehicle for 72 h. A part of P6 utricles were first infected with AdcD1, pulsed with EdU for 24 h between days 2 and 3, and thereafter treated with DAPT for 72 h, followed by fixation at 6 DIV. DAPT was dissolved in DMSO (concentration in the medium 0.1%) and used at the concentration of 10 µM. The compounds were added to the culture medium every 24 h.

### Irradiation of whole mount specimens

Utricular (P6 and P50) explants were irradiated with 2 Gy using calibrated ^137^Cs γ-ray source (BioBeam 8000, STS GmbH). Utricles were cultured thereafter for 1 or 4 h.

### Imaging

Histological cross-sections as well as wholemount surface preparations were analyzed under Axio Imager.M2 microscope (Zeiss) using brightfield and epifluorescence optics. Images were acquired through CCD colour camera (AxioCam HRc, Zeiss) and ZEN software (Zeiss), and processed using Adobe Photoshop CS6 (Adobe Systems). Confocal images were acquired using a Leica TCS SP5 microscope with Plan Apochromat 63×/1.3 NA objective. The acquisition software was Leica LAS AF. Z-projections were processed with Imaris 7 (Bitplane Scientific Software). Blind 3D deconvolution was made for Z-projections with AutoQuant X2 (Media Cybernetics).

### Quantification

To quantify the amount of adenovirus-infected utricular hair cells, parvalbumin+/GFP+ hair cells were counted from one random 40x microscopic field per AdGFP-infected P6 utricular explant at 3 DIV, each field covering striolar and non-striolar areas. Data are shown as the percentage of parvalbumin+/GFP+ hair cells (mean ± SEM).

To quantify the amount of proliferating cells following AdcD1 infection, Sox2+ and EdU+/Sox2+ SCs were counted from one random 40x microscopic field per infected P6 and P50 utricular explant at 3 DIV, each field covering striolar and non-striolar areas. Data are shown as the percentage of EdU+/Sox2+ SCs out of Sox2+ SCs (mean ± SEM).

To quantify the resolution of γH2AX+ foci, EdU+ and EdU+/γH2AX foci+ SCs were counted from one random 40x microscopic field per AdcD1-infected P6 and P50 utricular explant at 3, 7 and 14 DIV, each field covering striolar and non-striolar areas. Data are shown as the average percentage of EdU+/γH2AX+ cells out of EdU+ SCs (mean ± SEM).

To determine the relative proportion of SCs with pan-nuclear γH2AX profiles, Sox2+/ total γH2AX+ (foci plus pan-γH2AX) and Sox2+/pan-γH2AX+ SCs were counted from one random 40x microscopic field per P6 and P50 utricle at 7 DIV, each field covering striolar and non-striolar areas. Data are shown as the relative numbers of Sox2+/pan-γH2AX+ SCs out of Sox2+/total γH2AX+ SCs (mean ± SEM).

To determine the relative proportion of γH2AX+/Rad51- SCs, γH2AX+/Rad51+ and γH2AX+/Rad51- cells were counted from one random 40x microscopic field per P6 and P50 AdcD1-infected utricle at 3, 7 and 14 DIV, each field covering striolar and non-striolar areas. Data are shown as the percentage of Rad51+/γH2AX- SCs out of Rad51+/γH2AX+ SCs (mean ± SEM).

To quantify the amount of EdU+ hair cells following AdcD1-infection alone and in combination with DAPT treatment, EdU+/myo6+ cells were counted from one random 40x microscopic field per P6 AdcD1-infected and, AdcD1-infected and DAPT-treated utricle at 6 DIV, each field covering striolar and non-striolar areas. Data are shown as the total number of myo6+ and EdU+/myo6+ cells.

Three or five individual explants (*n*) were used for each quantification. Two-tailed Student's t test and one-way ANOVA were used for statistical analysis. A P-value ≤0.005 was considered highly significant.
